# Nocturnal heart rate variability may be useful for determining the efficacy of mandibular advancement devices for obstructive sleep apnea

**DOI:** 10.1038/s41598-020-57780-7

**Published:** 2020-01-23

**Authors:** Jeong-Whun Kim, Sung Ok Kwon, Woo Hyun Lee

**Affiliations:** 1 Department of Otorhinolaryngology, Seoul National University College of Medicine, Seoul National University Bundang Hospital, Seongnam, Republic of Korea; 2 Biomedical Research Institute, Kangwon National University Hospital, Chuncheon, Republic of Korea; 3 Department of Otolaryngology, Kangwon National University Hospital, Chuncheon, Republic of Korea

**Keywords:** Sleep disorders, Medical research

## Abstract

A mandibular advancement device (MAD) is recommended as an alternative therapy for obstructive sleep apnea (OSA), which effectively reduces the collapsibility of the upper airway during sleep by advancing the mandible. However, the effects of MAD therapy on cardiac autonomic modulation remain unclear. We evaluated the effects of MAD on nocturnal heart rate variability (HRV) in OSA. Anthropometric data, questionnaire results, and HRV parameters (evaluated using time domain and frequency domain methods) of 58 adult patients with OSA treated with MAD therapy were retrospectively reviewed. All patients underwent polysomnography at baseline and 3-month follow-up. The average normal-to-normal (NN) interval, standard deviation of the NN interval, low-frequency power in normalized units (LFnu), and high-frequency power in normalized units (HFnu) showed significant changes with MAD therapy. Based on the criteria for success (decrease in the apnea-hypopnea index by >50% and value <20/h), 34 and 24 patients were classified into the response and nonresponse groups, respectively. No differences in baseline characteristics were detected between groups, except for higher body mass index and lower minimal oxygen saturation in the nonresponse group. A subgroup analysis indicated that the average NN interval and HFnu significantly increased, and that Total power (TP), very low frequency, low frequency(LF), low frequency/high frequency and LFnu significantly decreased compared to baseline in the response group; however, no HRV changes were found in the nonresponse group. After adjusting for age, sex, and body mass index, the response group showed significant changes from baseline in TP and LF compared to the nonresponse group. Therefore, HRV may be useful for determining the efficacy of MAD therapy in OSA.

## Introduction

Obstructive sleep apnea (OSA) is characterized by repetitive upper airway obstruction during sleep associated with arterial blood desaturation, sympathetic nervous system activation, and cardiovascular impairment^1^. Untreated patients with OSA have increased mortality rates compared to the general population^2,3^. Laboratory polysomnography (PSG) is accepted as a standard method of diagnosing OSA^4^. However, PSG can be cumbersome because of its time, space, equipment, and tester requirements.

Heart rate variability (HRV) measures the variation in the time interval between heartbeats and reflects cardiac sympathetic and parasympathetic modulation. It can be measured using the single-lead electrocardiography (ECG) signal and is also called cycle length variability, RR variability, or heart period variability^5^. Reduced HRV was found to be related to increased mortality after myocardial infarction, congestive heart failure, diabetic neuropathy, depression, and poor survival in premature infants^5–9^. Because patients with OSA show abnormal cardiac autonomic modulation, the association between OSA and changes in HRV has been reported by several studies^10–13^.

A mandibular advancement device (MAD) is an oral device that has been recommended as an alternative therapy for OSA^14^. It can effectively reduce the collapsibility of the upper airway during sleep by advancing the mandible^15^. MAD was found to have positive effects on cardiac autonomic modulation measured by HRV in OSA that are similar to those of other treatments such as continuous positive airway pressure (CPAP) therapy^16–18^. However, there is still insufficient evidence for HRV changes occurring with successful MAD therapy for OSA. Furthermore, the positive effects of MAD on cardiac autonomic modulation are less clear than those of CPAP. Therefore, studies of the effects of MAD on cardiac morbidities are warranted. We attempted to identify whether MAD can affect cardiac autonomic modulation during an entire night. Our hypothesis was that a positive treatment response to MAD therapy may be associated with positive changes in HRV. This study evaluated the effects of MAD on HRV changes according to the response of treatment in patients with OSA.

## Results

### Changes in polysomnography statistics

PSG parameters related to the respiratory index were significantly improved by MAD when compared to baseline. MAD treatment significantly decreased the apnea-hypopnea index (AHI), apnea index (AI), hypopnea index (HI), and oxygen desaturation index (ODI). The minimal and average oxygen saturations significantly increased; however, the snoring rates were not changed with MAD. The incidence of sleep stage N3 and REM sleep significantly increased from 4.8 ± 5.5% to 7.2 ± 6.8% (*P* < 0.001) and from 15.6 ± 5.8% to 18.5 ± 6.2% (*P* < 0.001), respectively (Table 1).

**Table 1:** Laboratory full-night polysomnographic parameters without and with the use of a mandibular advancement device for patients with obstructive sleep apnea.

	Baseline	With MAD	*P* value
**Sleep parameters**			
Total sleep time, min	384.2 ± 55.0	389.2 ± 58.0	0.627
WASO, min	69.4 ± 40.8	59.1 ± 36.9	0.076
Sleep latency, min	15.8 ± 24.8	11.5 ± 9.6	0.152
Sleep efficiency, %	81.8 ± 9.9	83.1 ± 13.1	0.448
Arousal, per hour	31.5 ± 21.0	11.3 ± 12.8	<0.001^*^
**Sleep stage, % of total sleep time**			
N1	16.0 ± 7.9	12.2 ± 18.0	0.132
N2	48.6 ± 10.3	51.8 ± 9.9	0.077
N3	4.8 ± 5.5	7.2 ± 6.8	<0.001^*^
REM sleep	15.6 ± 5.8	18.5 ± 6.2	<0.001^*^
**Respiratory index**			
AHI, per hour	41.0 ± 20.1	19.6 ± 17.1	<0.001^*^
Hypopnea index, per hour	16.1 ± 7.5	11.8 ± 8.9	0.001^*^
ODI, per hour	30.6 ± 20.7	12.8 ± 15.0	<0.001^*^
Minimal oxygen saturation, %	80.1 ± 6.9	83.4 ± 6.3	<0.001^*^
Average oxygen saturation, %	94.8 ± 1.8	95.5 ± 1.5	<0.001^*^
Snoring, %	33.0 ± 18.2	30.1 ± 18.9	0.287

MAD, mandibular advancement device; WASO, wake time after sleep onset; REM, rapid eye movement; AHI, apnea-hypopnea index; ODI, oxygen desaturation index. ^*^P < 0.05.

### Changes in nocturnal heart rate variability

MAD treatment resulted in changes in HRV. Among the time domain measures, the average normal-to-normal (NN) interval significantly increased from 949.3 ± 134.1 ms to 988.4 ± 127.0 ms (*P* = 0.001), and the standard deviation of the NN interval (SDNN) significantly decreased from 96.8 ± 32.6 ms to 87.4 ± 26.8 ms (*P* = 0.042). Regarding the frequency domain values, the low-frequency (LF) power in normalized units (LFnu) and high-frequency (HF) power in normalized units (HFnu) showed statistically significant changes. LFnu decreased from 70.5 ± 11.9 to 67.4 ± 13.8 (*P* = 0.022) and HFnu increased from 29.5 ± 11.9 to 32.6 ± 13.8 (*P* = 0.022) with the use of MAD (Table 2).

**Table 2:** Changes in nocturnal heart rate variability after the use of a mandibular advancement device for patients with obstructive sleep apnea.

Variable	Baseline	With MAD	*P* value
**Time domain measures**			
Average NN interval, ms	949.3 ± 134.1	988.4 ± 127.0	0.001*
SDNN, ms	96.8 ± 32.6	87.4 ± 26.8	0.042*
SDANN, ms	65.2 ± 30.7	67.6 ± 43.2	0.728
RMSSD, ms	58.3 ± 45.5	48.4 ± 36.8	0.140
NN50 count	3,089 ± 3,175	2,860 ± 2,944	0.519
pNN50, %	11.2 ± 11.2	11.0 ± 11.8	0.839
HRV triangular index	16.5 ± 5.0	16.0 ± 4.3	0.473
**Frequency domain measures**			
Total power, ms^2^	51,905 ± 25,730	48,241 ± 25,666	0.243
VLF, ms^2^	29,058 ± 18,160	26,480 ± 17,101	0.166
LF, ms^2^	15,884 ± 7,984	14,929 ± 9,359	0.320
HF, ms^2^	5,790 ± 2,619	6,140 ± 2,720	0.310
LF/HF ratio	3.0 ± 1.9	2.8 ± 2.0	0.242
LFnu	70.5 ± 11.9	67.4 ± 13.8	0.022*
HFnu	29.5 ± 11.9	32.6 ± 13.8	0.022*

MAD, mandibular advancement device; SDNN, standard deviation of NN intervals; SDANN, standard deviation of the 5-min averages of NN intervals; RMSSD, the square root of the mean of the squared differences of adjacent NN intervals; NN50 count, number of pairs of adjacent NN intervals more than 50 ms; pNN50, rate of NN50 in the total number of NN intervals; VLF, very low frequency; LF, low frequency; HF, high frequency; LFnu, LF power in normalized units; HFnu, HF power in normalized units. ^*^P < 0.05.

### Subgroup analyses of response and nonresponse groups

According to the response criteria, 34 subjects were classified into the response group and 24 were classified into the nonresponse group. No significant differences in baseline characteristics were found between the two subgroups except for higher body mass index (*P* = 0.004) and lower minimal oxygen saturation (*P* = 0.048) in the nonresponse group (Table 3). However, significant HRV differences were detected between the two groups. Regarding the time domain measures, the average NN interval significantly increased in the response group (from 947.7 ± 152.0 ms to 998.9 ± 140.0 ms; *P* = 0.003); however, no significant difference was found in the nonresponse group (from 951.8 ± 106.6 ms to 973.6 ± 107.2 ms; *P* = 0.111). Other time domain variables did not significantly change in both groups. Among the frequency domain values, total power (TP, *P* = 0.007), very low frequency (VLF, *P* = 0.010), LF (*P* = 0.004), LF/HF (*P* = 0.031), and LFnu (*P* = 0.015) significantly decreased in the response group, but not in the nonresponse group. There was also a significant change in HFnu in the response group (from 28.8 ± 10.1 to 33.4 ± 12.6; *P* = 0.015), but not in the nonresponse group (from 30.6 ± 14.3 to 31.3 ± 14.3; *P* = 0.686). The remaining frequency domain variable (HF) did not change with MAD in either group (Table 4). According to the success criteria of AHI that decreased by more than 50% and had a value less than 10/h, 24 subjects were classified to success group and 34 subjects were classified to failure group. In analyzing the change in HRV of each group, the average NN interval, TP, VLF, LF, LFnu, and HFnu significantly changed in the success group, while average NN interval and SDNN significantly change in the failure group (Table 5). Finally, the response group showed significant changes from baseline in the TP [−11004.00 (95% CI, −21448.00 to −559.85) in the response group and 5162.16 (95% CI, −7804.99 to 18129.00) in the nonresponse group; *P* = 0.02] and LF [−3483.21 (95% CI, −6690.23 to −276.19) and 2057.00 (95% CI, −1924.82 to 6038.81); *P* = 0.012] compared to the nonresponse group after adjusting for age, sex, and body mass index. However, changes in the sleep stage did not differ between the response and nonresponse groups (Table 6).

**Table 3:** Baseline characteristics of the treatment response and nonresponse groups.

	Response (N = 34)	Nonresponse (N = 24)	*P* value
Age, years	52.9 ± 9.7	53.3 ± 8.3	0.858
Male/female ratio	29/5	22/2	0.688
Body mass index, kg/m^2^	24.9 ± 3.0	27.4 ± 3.0	0.004^*^
Waist-to-hip ratio	0.93 ± 0.05	0.91 ± 0.19	0.639
AHI (per hour)	37.8 ± 15.7	45.4 ± 24.8	0.193
Apnea index, per hour	22.3 ± 16.7	28.7 ± 24.1	0.269
Minimal oxygen saturation, %	81.6 ± 6.8	78.0 ± 6.6	0.048^*^
ODI, per hour	26.4 ± 16.2	36.5 ± 25.0	0.090
ESS	10.1 ± 4.8	10.6 ± 5.6	0.733
PSQI	6.4 ± 3.2	6.7 ± 3.7	0.793

MAD, mandibular advancement device; AHI, apnea-hypopnea index; ODI, oxygen desaturation index; ESS, Epworth sleepiness scale; PSQI, Pittsburgh sleep quality index. ^*^P < 0.05.

**Table 4:** Changes in nocturnal heart rate variability after the use of a mandibular advancement device in the response and nonresponse groups.

Variable	Response (N = 34)			Nonresponse (N = 24)		
	Baseline	With MAD	*P* value	Baseline	With MAD	*P* value
**Polysomnographic parameters**						
AHI, per hour	37.8 ± 15.7	9.6 ± 4.9	<0.001^*^	45.4 ± 24.8	33.7 ± 18.3	0.003^*^
Apnea index, per hour	22.3 ± 16.7	2.7 ± 2.9	<0.001^*^	28.7 ± 24.1	15.1 ± 16.2	<0.001^*^
Hypopnea index, per hour	15.6 ± 7.6	7.0 ± 3.6	<0.001^*^	16.8 ± 7.5	18.7 ± 9.6	0.302
ODI, per hour	26.4 ± 16.2	4.6 ± 3.4	<0.001^*^	36.5 ± 25.0	24.4 ± 17.3	0.006^*^
Minimal oxygen saturation, %	81.6 ± 6.8	84.9 ± 7.0	0.001^*^	78.0 ± 6.6	81.3 ± 4.5	0.002^*^
Average oxygen saturation, %	95.3 ± 1.6	96.0 ± 1.5	0.010^*^	94.0 ± 1.8	94.8 ± 1.1	0.021^*^
**Sleep stage, % of total sleep time**						
N1	15.2 ± 7.9	11.6 ± 23.1	0.391	17.2 ± 8.0	12.8 ± 6.0	0.007^*^
N2	47.9 ± 9.3	52.7 ± 9.9	0.047^*^	49.6 ± 11.7	50.0 ± 10.2	0.894
N3	6.0 ± 6.0	7.3 ± 6.1	0.114	3.1 ± 4.2	7.1 ± 7.8	0.003^*^
REM sleep	15.2 ± 6.6	19.2 ± 6.2	0.001^*^	16.1 ± 4.7	17.3 ± 6.3	0.378
**Time domain measures**						
Average NN interval, ms	947.7 ± 152.0	998.9 ± 140.0	0.003^*^	951.8 ± 106.6	973.6 ± 107.2	0.111
SDNN, ms	98.0 ± 34.7	89.9 ± 25.1	0.175	95.0 ± 30.1	84.0 ± 29.2	0.136
SDANN, ms	61.4 ± 26.2	76.6 ± 48.1	0.075	70.5 ± 36.0	54.7 ± 32.0	0.137
RMSSD, ms	55.1 ± 46.1	45.6 ± 25.7	0.184	62.8 ± 45.2	52.5 ± 48.7	0.421
NN50 count	2,998 ± 2,882	2,639 ± 2,634	0.449	3,218 ± 3,611	3,172 ± 3,370	0.933
pNN50, %	11.0 ± 10.4	10.4 ± 11.2	0.760	11.7 ± 12.5	11.8 ± 12.9	0.962
HRV triangular index	16.7 ± 5.5	16.1 ± 4.5	0.513	16.2 ± 4.4	16.0 ± 4.1	0.767
**Frequency domain measures**						
Total power, ms^2^	54,810 ± 26,689	45,169 ± 20,785	0.007^*^	47,789 ± 24,260	52,594 ± 31,288	0.385
VLF, ms^2^	31,706 ± 19,169	24,731 ± 13,606	0.010^*^	26,393 ± 16,517	28,960 ± 21,171	0.475
LF, ms^2^	16,487 ± 8,011	13,616 ± 7,906	0.004^*^	15,031 ± 8,036	16,790 ± 11,010	0.335
HF, ms^2^	5,871 ± 2,644	6,153 ± 2,589	0.523	5,676 ± 2,635	6,121 ± 2,954	0.431
LF/HF ratio	3.1 ± 2.0	2.5 ± 1.5	0.031^*^	2.9 ± 1.7	3.2 ± 2.5	0.331
LFnu	71.2 ± 10.1	66.6 ± 12.6	0.015^*^	69.4 ± 14.3	68.7 ± 14.3	0.686
HFnu	28.8 ± 10.1	33.4 ± 12.6	0.015^*^	30.6 ± 14.3	31.3 ± 14.3	0.686

MAD, mandibular advancement device; AHI, apnea-hypopnea index; REM, rapid eye movement; ODI, oxygen desaturation index; REM, rapid eye movement; SDNN, standard deviation of NN intervals; SDANN, standard deviation of the 5-min averages of NN intervals; RMSSD, the square root of the mean of the squared differences of adjacent NN intervals; NN50 count, number of pairs of adjacent NN intervals more than 50 ms; pNN50, rate of NN50 in total number of NN intervals; VLF, very low frequency; LF, low frequency; HF, high frequency; LFnu, LF power in normalized units; HFnu, HF power in normalized units. ^*^P < 0.05.

**Table 5:** Changes in nocturnal heart rate variability after the use of a mandibular advancement device in the success and failure groups.

Variable	Success (N = 24)			Failure (N = 34)		
	Baseline	With MAD	*P* value	Baseline	With MAD	*P* value
**Time domain measures**						
Average NN interval, ms	968.4 ± 136.3	1004.8 ± 130.0	0.031^*^	935.9 ± 132.9	976.9 ± 125.5	0.010^*^
SDNN, ms	95.3 ± 33.6	89.0 ± 24.4	0.447	97.8 ± 32.4	86.3 ± 28.7	0.035^*^
SDANN, ms	62.3 ± 24.7	71.6 ± 36.2	0.213	67.3 ± 34.5	64.7 ± 47.9	0.799
RMSSD, ms	52.0 ± 49.2	41.6 ± 22.8	0.293	62.7 ± 42.9	53.2 ± 43.8	0.302
NN50 count	2,545 ± 2,913	2,248 ± 2,210	0.615	3,473 ± 3,337	3,291 ± 3,332	0.688
pNN50, %	9.2 ± 10.0	8.4 ± 8.4	0.686	12.7 ± 11.9	12.8 ± 13.5	0.929
HRV triangular index	15.8 ± 4.6	15.4 ± 4.6	0.746	17.0 ± 5.3	16.5 ± 4.1	0.507
**Frequency domain measures**						
Total power, ms^2^	54,330 ± 26,087	44,123 ± 18,974	0.017^*^	50,193 ± 25,727	51,148 ± 29,426	0.828
VLF, ms^2^	31,474 ± 19,753	24,367 ± 12,701	0.032^*^	28,120 ± 17,113	27,973 ± 19,677	0.960
LF, ms^2^	16,324 ± 7,501	13,156 ± 7,487	0.008^*^	15,574 ± 8,405	16,181 ± 10,407	0.664
HF, ms^2^	5,822 ± 2,292	5,960 ± 2,482	0.796	5,768 ± 2,861	6,266 ± 2,907	0.278
LF/HF ratio	3.1 ± 2.2	2.5 ± 1.6	0.083	3.0 ± 1.6	3.0 ± 2.2	0.801
LFnu	71.2 ± 10.4	66.8 ± 11.2	0.049^*^	69.9 ± 13.0	67.9 ± 15.5	0.214
HFnu	28.8 ± 10.4	33.2 ± 11.2	0.049^*^	30.1 ± 13.0	32.1 ± 15.5	0.214

MAD, mandibular advancement device; SDNN, standard deviation of NN intervals; SDANN, standard deviation of the 5-min averages of NN intervals; RMSSD, the square root of the mean of the squared differences of adjacent NN intervals; NN50 count, number of pairs of adjacent NN intervals more than 50 ms; pNN50, rate of NN50 in total number of NN intervals; VLF, very low frequency; LF, low frequency; HF, high frequency; LFnu, LF power in normalized units; HFnu, HF power in normalized units. ^*^P <0.05.

**Table 6:** Comparison of changes from baseline in nocturnal heart rate variability after the use of a mandibular advancement device between the response and nonresponse groups.

Variable	Response (N = 36)	Nonresponse (N = 22)	*P* value
**Time domain measures**			
Average NN interval, ms	44.48 (6.02 to 82.94)	−0.37 (−48.12 to 47.38)	0.086
SDNN, ms	−8.78 (−25.02 to 7.47)	−3.75 (−23.92 to 16.42)	0.645
SDANN, ms	23.84 (1.30 to 46.39)	−1.21 (−29.21 to 26.79)	0.101
RMSSD, ms	−7.34 (−31.41 to 16.73)	−2.20 (−32.08 to 27.68)	0.750
NN50 count	25.82 (−1263.01 to 1314.66)	367.47 (−1232.73 to 1967.68)	0.692
pNN50, %	1.01 (−3.59 to 5.61)	1.93 (−3.79 to 7.64)	0.766
HRV triangular index	−1.16 (−3.39 to 1.07)	−0.08 (−2.85 to 2.70)	0.470
**Frequency domain measures**			
Total power, ms^2^	−11004.00 (−21448.00 to −559.85)	5162.16 (−7804.99 to 18129.00)	0.024^*^
VLF, ms^2^	−7695.10 (−15021.00 to −368.97)	2580.36 (−6515.71 to 11676.00)	0.061
LF, ms^2^	−3483.21 (−6690.23 to −276.19)	2057.00 (−1924.82 to 6038.81)	0.012^*^
HF, ms^2^	197.72 (−1049.70 to 1445.13)	404.02 (−1144.76 to 1952.80)	0.805
LF/HF ratio	−0.55 (−1.25 to 0.16)	0.33 (−0.55 to 1.21)	0.069
LFnu	−4.53 (−9.13 to 0.07)	−1.28 (−7.00 to 4.43)	0.295
HFnu	4.53 (−0.07 to 9.13)	1.28 (−4.43 to 7.00)	0.295
**Sleep stage, % of total sleep time**			
N1	−6.41 (−15.18 to 2.36)	−5.94 (−16.83 to 4.95)	0.937
N2	2.21 (−4.23 to 8.65)	−2.98 (−10.98 to 5.01)	0.231
N3	2.78 (0.34 to 5.23)	5.12 (2.08 to 8.15)	0.157
REM sleep	3.66 (1.08 to 6.25)	1.18 (−2.03 to 4.39)	0.156

MAD, mandibular advancement device; SDNN, standard deviation of NN intervals; SDANN, standard deviation of the 5-min averages of NN intervals; RMSSD, the square root of the mean of the squared differences of adjacent NN intervals; NN50 count, number of pairs of adjacent NN intervals more than 50 ms; pNN50, rate of NN50 in total number of NN intervals; VLF, very low frequency; LF, low frequency; HF, high frequency; LFnu, LF power in normalized units; HFnu, HF power in normalized units; REM, rapid eye movement.

Values are reported as least squares means (LS-means) and the 95% confidence intervals from the ANCOVA adjusted for age, sex, and body mass index. ^*^P < 0.05.

## Discussion

This study revealed that MAD treatment significantly changed cardiac autonomic modulation represented by nocturnal HRV and that the changes were significant only in the treatment response group. MAD reduces AHI less effectively than CPAP, but it has been associated with higher compliance^19^; therefore, it has been widely used as an alternative treatment for patients with OSA. However, few studies have assessed the effects of MAD on cardiac autonomic modulation. A previous study that included 10 patients with OSA treated with MAD showed significant changes in the NN interval, HF, and the LF/HF ratio^16^. However, our findings showed significant changes in the NN interval, SDNN, LFnu, and HFnu. This discrepancy could be related to the difference in the number of subjects (10 vs. 58), HRV measurement time (day vs. night), and the treatment success rate (100% success group vs. 62.1% response group). Two previous studies that compared CPAP and MAD also described changes in HRV after MAD treatment. One study showed that MAD significantly decreased TP compared to baseline, and that the decrease was greater than that resulting from the use of a placebo oral appliance but lower than that resulting from CPAP^17^. Another study found no difference between MAD and CPAP regarding cardiac autonomic function changes during the day, although CPAP was more effective than MAD for eliminating respiratory events^18^. Although these previous comparative studies showed that MAD treatment induced significant HRV changes, their results were limited by the relatively small numbers of subjects (29 and 40) and restricted numbers of parameters that were used to assess HRV. Because we included 58 subjects and evaluated most HRV parameters, our results are likely to provide more concrete evidence regarding the effects of MAD on HRV. However, the difference in HRV changes was weakened between success and failure groups. HRV significantly changed on average NN interval, TP, VLF, LF, LFnu, and HFnu in the success group, while changed on average NN interval and SDNN in the failure group. Considering the average AHI (41.0/h) of our study subjects, the success criteria of AHI (decreased by more than 50% and had a value less than 10/h) was too strict. Accordingly, some subjects who had a good therapeutic effect on MAD were classified to failure group could be a reason for weakened difference between success and failure groups.

HRV varies considerably between individuals and is affected by age and physical condition^20^. Therefore, there is no uniform definition of the normal HRV range, and it is more difficult to determine a cutoff value for diagnosing OSA. Previous studies have reported the results of various HRV parameters for the diagnosis of OSA through various research methods. One previous study presented the LF/HF ratio as a useful parameter for diagnosing OSA in correlation with AHI^13^. Another study that examined the difference between HRV during the day and that during the night found that day and night SDNN were able to screen for OSA with high sensitivity and specificity^21^. Another study suggested that the NN interval was the best index among the time and frequency domain parameters because only the mean NN interval was shorter in the OSA group than in the control group^22^. As previously mentioned, studies that used HRV to diagnose OSA showed generally inconsistent results. These different reasons were due to the lack of uniform study methods and HRV characteristics that varied with age and physical condition. Therefore, to clarify the relationship between OSA and HRV, a well-designed research method is needed.

It seems that HRV is more useful for determining treatment results than for diagnosing OSA. A few studies have reported changes in HRV after OSA treatment. One study on changes in HRV after CPAP therapy reported that the SDNN of non-REM sleep decreased after CPAP^23^. We also found that the SDNN was decreased regardless of the sleep stage after MAD treatment. However, it was impossible to determine the response or nonresponse to treatment using the SDNN. A decrease in the LF/HF ratio was reported for 18 children after adenotonsillectomy^24^. We also found a change in the LF/HF ratio with response group of MAD therapy. Despite the differences in the age of the subjects (children vs. adults) and treatment modality (adenotonsillectomy vs. MAD) between studies, the change in the LF/HF ratio was similar. Choi *et al*.^25^ concluded that HRV showed significant changes in the success group of OSA patients who underwent upper airway surgery, but not in the failure group of OSA patients. They reported that the changes in VLF, LF, LFnu, and HFnu were meaningful, and these findings were in partial agreement with our results. We found that TP, VLF, LF, LFnu, and HFnu showed significant changes among the frequency domain parameters in the response group.

HRV is easier and less expensive to evaluate than PSG. The final model of our study showed that the TP and LF significantly changed in the response group compared to those in the nonresponse group after adjusting for age, sex, and body mass index. Taken together, changes in HRV, including decreased TP and decreased LF, could be used to determine the response of MAD treatment.

Previous studies that have analyzed the changes in HRV and evaluated treatment success have not included time domain paraments^25^ or only described the results of short-term analyses^23^. However, after analyzing the time domain parameters during 5 night h according to the standard method, we found that the NN interval had significantly changed in the response group, which was a strength of our study.

This study had several limitations. First, various success criteria were applied in different studies. Among many definitions of OSA treatment success, we used commonly accepted criteria, namely more than a 50% decrease in AHI and a value less than 20/h with MAD treatment. Therefore, the results of this study might differ from those of studies that used other success criteria. Second, the time domain method was applied during 5 h during the night. The HRV Task Force has indicated that it seems appropriate to analyze time domain method results using nominal 24-h long-term recordings^5^. To overcome this limitation, we used recordings of ECG during the same 5-h periods to analyze the time domain method results according to alternative recommendations. Third, selection bias could not be excluded because patients with better outcomes were more likely to participate in follow-up testing. A prospective study is needed to validate the effects of MAD treatment on cardiac autonomic modulation in OSA. Fourth, the sleep stage may influence the HRV values. However, in this study, we did not perform a detailed analysis of HRV according to sleep stage. Instead, we found that changes in the sleep stage did not differ between the response and nonresponse groups. Therefore, the sleep stage may have had a minor effect on the differences in HRV values between groups. Nevertheless, follow-up studies of HRV according to the sleep stage are needed.

In conclusion, the present study showed that MAD treatment for OSA significantly changed cardiac autonomic modulation as represented by nocturnal HRV. Among the assessed parameters, the average NN interval and HFnu increased, while TP, VLF, LF, LF/HF ratio, and LFnu decreased. However, the changes were observed only in the response group. Moreover, after adjusting for age, sex, and body mass index, the response group showed significant changes in the TP and LF compared to the nonresponse group. Therefore, nocturnal HRV may be a useful screening tool for determining the efficacy of MAD.

## Methods

### Subjects

**Figure 1:**
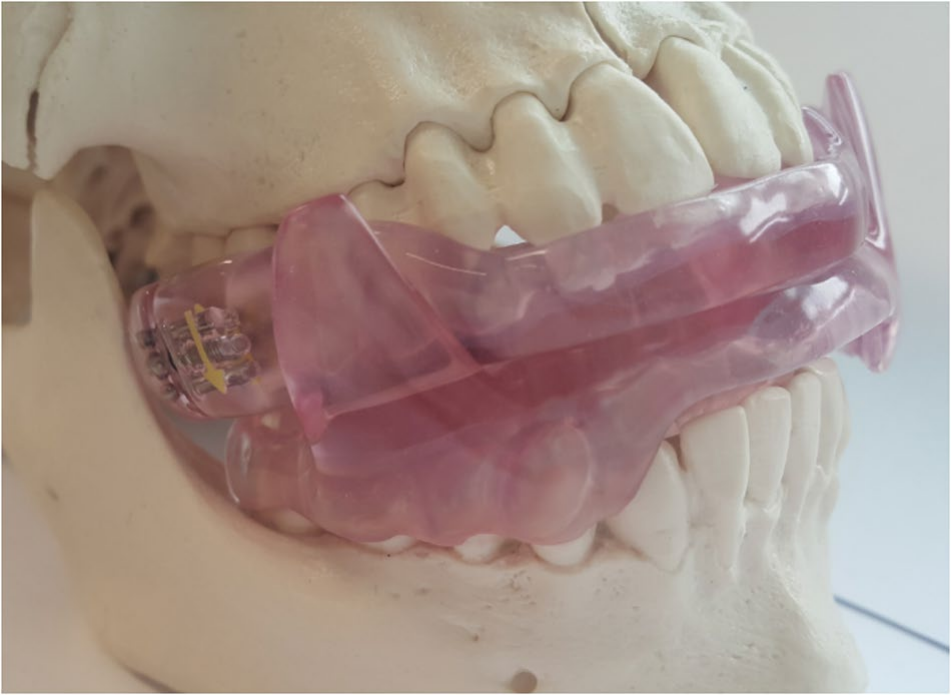
Mandibular advancement device.

We retrospectively reviewed the data of patients who visited a sleep center of a tertiary hospital because of snoring and sleep apnea. We selected subjects according to inclusion and exclusion criteria. The inclusion criteria were as follows: (1) adult patients (age ≥ 18 years); (2) patients who were diagnosed with OSA (AHI > 5/h); (3) patients who were treated with MAD (SomnoDent; SomnoMed Ltd, New South Wales, Australia) (Fig. 1); and (4) patients who underwent follow-up PSG after 3 months of MAD therapy and had the results of two PSG tests (baseline and with MAD) available. The exclusion criteria were the follows: (1) significant arrhythmias; (2) low-quality data (artefact > 20% of total sleep time); (3) total sleep time less than 5 h; (4) awake more than 30 minutes from midnight to 5 am; (5) combined sleep disorders (i.e., insomnia or narcolepsy); (6) habitual use of sedatives and hypnotics; and (7) history of specific pathology related to HRV changes (i.e., myocardial infarction, diabetic neuropathy, cardiac transplantation, myocardial dysfunction, or tetraplegia). Based on the inclusion and exclusion criteria, a total of 58 patients with OSA were included in this study. The anthropometric data of subjects were evaluated. Daytime sleepiness and subjective sleep quality of subjects were evaluated using the Epworth sleepiness scale (ESS) and Pittsburgh sleep quality index (PSQI), respectively. The ethics committee of Seoul National University Bundang Hospital (IRB No. B-1902-522-111) approved the use of these data. The need for written informed consent was waived by the Institutional Review Board.

### Analyses of nocturnal heart rate variability

The HRV was measured using exported ECG data and commercially available PSG software (RemLogic 3.0 HRV analyzer; Embla Systems, San Carlos, CA). The HRV analysis was performed without any information regarding PSG results except for the ECG signal. To prevent inappropriate comparisons, we analyzed the ECG signal from midnight to 5 am. In other words, ECG signals exported before midnight and after 5 am were not included in the HRV analysis. The signals were interpolated and resampled at 5.0 Hz; NN intervals more than 2,400 ms and less than 400 ms were omitted.

Calculations of the time domain and frequency domain parameters were performed according to the standard methods for HRV measurements^5^. The following parameters measured using time domain methods were included: (1) average NN interval; (2) SDNN; (3) standard deviation of the 5-minute averages of NN intervals (SDANN); (4) square root of the mean of the squared differences of adjacent NN intervals (RMSSD); (5) number of pairs of adjacent NN intervals more than 50 ms (NN50 count); (6) the rate of NN50 in the total number of NN intervals (pNN50); and (7) HRV triangular index and geometric measurements. Frequency domain variables were presented as the average of the values calculated every 5 minutes. The parameters of the frequency domain and their brief descriptions were as follows: total power, power of approximately ≤ 0.4 Hz; VLF, power ≤ 0.04 Hz; LF, power of 0.04–0.15 Hz; HF, power of 0.15–0.4 Hz; LF/HF ratio, LF divided by HF; LFnu, LF power of normalized units [LF/(LF + HF) × 100]; and HFnu, HF power of normalized units [HF/(LF + HF) × 100].

### Definition of treatment outcomes

Apnea was defined as the complete cessation of airflow for at least 10 s. Hypopnea was defined as a substantial reduction in airflow (≥30%) for at least 10 s associated with electroencephalographic arousal or oxygen desaturation (≥3%). AHI was defined as the total number of apnea incidents and hypopnea incidents per hour of sleep. Snoring was measured with a snore sensor (Piezo; Pro-Tech, Woodinville, Washington, USA). The sensor was attached around the neck of patients at the larynx level and detected whether vibration of the larynx was sufficient. The snoring rate was defined as the ratio of snoring time to total sleep time. The response to treatment was defined as an AHI that decreased by more than 50% and had a value less than 20/h with MAD compared to baseline^26^. Subjects who did not meet these criteria were included in the nonresponse group. The success to treatment was defined as an AHI that decreased by more than 50% and had a value less than 10/h with MAD compared to baseline. Subjects who did not meet these criteria were included in the failure group.

### Statistical analysis

Most values obtained during this study were continuous variables expressed as mean ± standard deviation, except for sex, which was a categorical variable presented as a ratio. The paired *t* test was used to compare the differences between the baseline values of parameters and those after MAD. The independent *t* test or chi-squared test was performed for the comparative analysis of baseline characteristics of the response and nonresponse groups. Differences in the change from baseline between the response and nonresponse groups were tested using an analysis of covariance (ANCOVA) with age, sex, and body mass index as covariates. Data analyses were performed using SPSS software (version 18; SPSS Inc., Chicago, IL), and *P* < 0.05 was considered statistically significant.

## Data Availability

The data that support the findings of this study are available from Seoul National University Bundang Hospital Sleep Center, but restrictions apply to the availability of these data, which were used under license for the current study; therefore, they are not publicly available. However, data are available from the authors upon reasonable request and with the permission of Seoul National University Bundang Hospital Sleep Center.
